# Decision-making for community resilience: A review of decision support systems and their applications

**DOI:** 10.1016/j.heliyon.2024.e33116

**Published:** 2024-06-14

**Authors:** Sahar Elkady, Josune Hernantes, Leire Labaka

**Affiliations:** Industrial Management Department, TECNUN, University of Navarra, Spain

**Keywords:** Community resilience, Systematic literature review, Decision support, Disaster management, Resilience operationalization

## Abstract

Decision Support Systems (DSS) have emerged as important tools for enhancing community resilience due to their ability to provide timely and efficient solutions to disaster-related problems while reflecting the perspectives of different stakeholders and utilizing multiple data sources. This paper provides a comprehensive summary of DSS applications to community resilience, emphasizing how the different modeling techniques are used in different disaster phases. We found that optimization techniques are the most frequently used methods for building DSS. Furthermore, we found that DSS tend to focus more on the preparedness and response phases of disaster management, rather than the recovery and mitigation phases. Moreover, the study highlights the main challenges in developing and implementing DSS for resilience, such as data availability, the uncertainty of the disaster context, and the need for cross-disciplinary collaboration.

Based on the reviewed papers, we provide some guidelines to practitioners to select the most suitable decision-support tools for the needs of their community. The study aims to help decision-makers and researchers build effective decision support systems for enhancing community resilience, considering the current challenges.

## 1 Introduction

The impacts of disasters have become increasingly severe; in the last five years, more people have died or been impacted by disasters than in the previous five years [1]. Moreover, the economic costs of disasters increased by 82 % between 1980-1999 and 2000–2019 [2], reaching $110 billion in losses during the first half of 2023 [3]. Additionally, climate change acts as a threat multiplier, intensifying existing disaster risks and introducing new ones. Risks and consequences linked to climate change span various domains, affecting health and wellness by exacerbating mental health concerns, infectious diseases, and malnutrition; cities and urban infrastructure through flooding and rising sea levels; and biodiversity and ecosystems through desertification, loss of biodiversity, land and forest degradation, and glacial retreat [4]. These increasing disaster impacts mandated the concept of community resilience to be included in disaster risk management strategies [5,6] due to its potential to provide tools for risk analysis and preparation, as well as deal with and recover from the consequences [7].

Disaster risk management (DRM) “is the application of disaster risk reduction policies and strategies to prevent new disaster risk, reduce existing disaster risk and manage residual risk, contributing to the strengthening of resilience and reduction of disaster losses” [8]. In this vein, Disaster risk reduction (DRR) “is the policy objective of disaster risk management” [8]. DRR strategies and policies focus on preventing new risks, reducing existing ones, and building resilience [8,9]. Resilience is then defined as “the ability of a system, community or society exposed to hazards to resist, absorb, accommodate, adapt to, transform and recover from the effects of a hazard in a timely and efficient manner, including through the preservation and restoration of its essential basic structures and functions through risk management.” [8]. There are synergies among these abilities and a tradeoff for their implementation [10].

According to the IPCC, DRM spans multiple phases of disaster management, prevention, preparedness, response, and recovery [9]. Resilience is woven into the fabric of DRM, playing a crucial role in all four phases. By proactively mitigating risks through prevention measures (e.g., building codes, flood control), communities experience a lessened overall impact from disasters. This translates to a greater ability to resist the initial shock and absorb the aftereffects, ultimately fostering a more resilient community. Preparedness further strengthens resilience by allowing communities to adapt to a disaster situation and a quicker recovery. During the response phase, swift and coordinated actions like search and rescue minimize loss of life and property damage, strengthening resilience by enabling communities to return to normalcy quickly. Finally, recovery processes that rebuild infrastructure and livelihoods are essential for long-term resilience. In essence, a strong DRM strategy that prioritizes all four phases paves the way for building resilience. This resilience, in turn, has the potential to decrease the impacts of unexpected events that are difficult to predict and manage.

The environment we live in nowadays is marked by complexity, interconnectedness, and perpetual change. Due to this complexity, risks have cascading non-linear impacts across multiple areas, which has introduced the concept of systemic risk. “*Systemic risk is based on the notion that the risk of an adverse outcome of a policy, action, or hazard event can depend on how the elements of the affected systems interact with each other. This can either aggravate or reduce the overall effect of the constituent parts.*” [1]. Enhancing community resilience is one of the recommendations to deal with systemic risks [11].

Community resilience is considered a "system of systems" [12] that encompasses various synergies among different resilience capabilities, since it is a combination of multiple interconnected systems, such as economic, social, environmental, and physical systems [10,13]. Given the interdependency between these systems and the cascading impacts of risks or enhancements from one system to another, it is hard to make decisions to reduce disaster risk impacts and enhance resilience, as we cannot consider one system in isolation from others. The uncertainty surrounding decision-making is further amplified by the presence of multiple data sources, stakeholders with diverse perspectives, varying decision-making capabilities, and different success measures and indicators [14]. Hence, a decision-making system is a must to provide the necessary tools for making effective decisions [15].

Decision support systems (DSS) provide decision-makers with analysis, information, recommendations, and environments to test different scenarios. Furthermore, DSS enables the operationalization of resilience. This is accomplished by developing analytical models that map the various operations and include all stakeholders [16], thus offering an in-depth understanding of what we mean by resilience and the whole resilience process. This adds to the body of knowledge in this field, as research on how to operationalize resilience is still limited [17]. Furthermore, DSS can contribute to more objective and evidence-based decision-making by mitigating the inherent subjectivity and normativity that can influence human analysis during the decision-making process [18]. Using DSS aligns with the UNDRR's focus on targeted and systematic resilience investments [19]. By employing DSS, decision-makers can ensure that scarce resources are strategically directed toward areas with the greatest need.

In this vein, several studies have explored the use of computational models in disaster management and resilience. For instance Ref. [20], provides a comprehensive overview of Artificial Intelligence (AI) applications in this domain. However, their focus on AI techniques necessitates data-intensive case studies, potentially overlooking less data-reliant modeling approaches or incorporating expert knowledge. Other studies address resilience, but their emphasis lies primarily on conceptualizing resilience frameworks and assessment models, or on dynamic modeling methods like game theory applications within the context of community resilience [21]. Additionally, some researchers have taken a narrower approach, examining the application of computational intelligence specifically in flood management [22]. There is a gap in the literature regarding studies that explore the general application of various DSS modeling techniques to community resilience. Hence, in this paper, we provide a comprehensive overview of the state of research on community resilience decision support systems, considering different modeling techniques, disaster phases, and hazard types. Moreover, we identify the gaps in the literature and provide a guideline for practitioners to select the most appropriate decision-support tools for the needs of their community.

The rest of this paper is structured as follows. Section 2 details the systematic literature review process employed, elaborating on the inclusion and exclusion criteria used to identify the relevant studies to this review. Additionally, the analysis process for the selected papers will be explained, concluding with a summary of the identified studies categorized by their modeling techniques. Section 3 delves into the results of the literature review, illuminating the current challenges and gaps within the existing research. Additionally, this section provides guidelines for selecting the most appropriate modeling technique based on the specific resilience problem under investigation. Finally, Section 4 presents the key conclusions drawn from the analysis.

## 2 Systematic literature review

### 2.1 Search approach

We searched the Web of Science (WoS) database for publications related to community resilience decision support using the following search queries: (community OR societal) AND resilience AND decision support AND (systems OR frameworks). We limited our search to articles in English published between December 2000 and December 2022.

Our focus being solely on decision support models that address community resilience, we utilized this specific set of keywords to investigate (1) which aspects of community resilience could be modeled, (2) which modeling techniques were used, and (3) how the stakeholders' involvement was considered. Our interest lies in understanding the modeling of community resilience, with a particular focus on the engagement of various stakeholders since improving resilience requires the involvement and collaboration of multiple stakeholders [23].

**Fig. 1:**
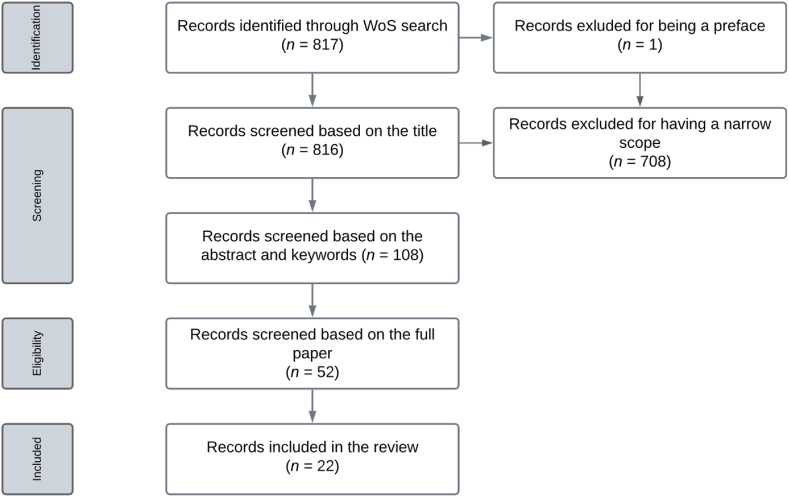
Literature review selection process.

To decide which papers to include in our review, we followed the PRISMA approach [24]. The PRISMA (Preferred Reporting Items for Systematic Reviews and Meta-Analyses) approach is a widely recognized and respected framework for conducting systematic literature reviews. It provides a comprehensive and transparent methodology, ensuring the clarity and rigor of the review process, from study identification to data synthesis. The review process outlined in Fig. 1 involved several steps. First, the previously mentioned search queries resulted in 817 records, none of which were repeated, and one we excluded, which was a preface. Second, we screened the titles of 816 publications. Papers were excluded if they cover a very narrow scope, for example, the resilience of fishery; or if they are related to specific groups in a community such as gays and transgender; or addressing ecological resilience or mental resilience. This screening resulted in 108 papers being selected for further review based on their abstracts and keywords, using the same exclusion criteria.

We also excluded studies that focus on the identification and evaluation of potential risks and their likelihood of occurrence since these studies do not focus on the mitigation of these risks nor the adaptive capacities needed to build resilience. Additionally, we excluded the studies that present assessment tools since they are measurement methods rather than modeling methods. This process resulted in 52 papers being fully read, of which 30 were eventually excluded due to factors such as not being a decision support system/framework, but just an evaluation model or an investigative model that studies a specific phenomenon and not directly oriented toward decision support. For example [25], presents a framework (KnoPE) to evaluate urban resilience knowledge products (such as models, tools, and assessments “designed to harness science and technology to link knowledge to action”). While valuable, KnoPE does not offer a decision support tool for building community resilience. Instead, it evaluates the knowledge products emphasizing context understanding, user communication, and knowledge product application, ultimately aiming to aid in the refinement of existing tools and the development of new effective tools. Excluding similar studies ensured our research focused on decision support systems directly applicable to building community resilience. Moreover, in Ref. [26] for instance, the authors presented a prototype for home reconstruction using Discrete Event Simulation (DES) to assess its potential for disaster recovery simulation. While the simulation lays the groundwork for more comprehensive models, the study focuses on developing the model using Python and its suitability for a specific software library (Simply), not on directly aiding decision-making during recovery efforts.

### 2.2 Analysis

**Fig. 2:**
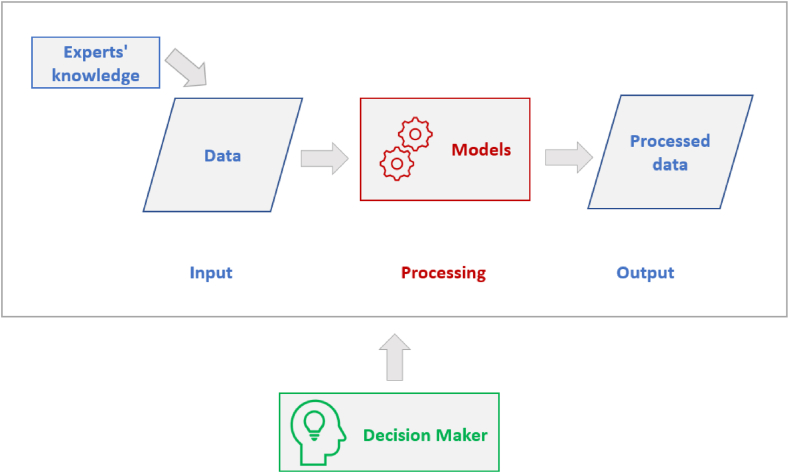
The main parts of a Decision Support Framework.

We based our analysis of the different publications on the general structure of a DSS [27] which consists of the components outlined in Fig. 2: (1) data component, which represents the input that needs to be processed. This data could come from a database, files, raw data, etc., and could be supported by experts’ knowledge, (2) models component, which includes the different types of computational models that are used to support the decision-making process based on the input data, (3) processed data, which represents the results produced by the models and the output of the whole system.

The processes across these three components are initiated and consumed by a decision-maker. Based on this structure we identified “modeling type” and “data sources” as two main criteria to characterize the identified publications. The decision-maker was omitted from our analysis as it is not prominently specified in many of the papers.

Moreover, we added more criteria for the analysis that are related to the resilience context, such as disaster phase, hazard type, and the location of the case study. Following is a description of each criterion.

- Modeling type: We aggregated the different modeling types into a higher category; for example, the various types of simulation modeling, such as System Dynamics, Agent-Based Modeling, and Monte Carlo simulation, are aggregated into the Simulation category. Table 1 shows all the modeling categories and subcategories used.

**Table 1:** Aggregation of modeling types.

Category	Subcategories
Simulation (S)	Monte Carlo simulation (MCS), Agent-Based Modeling (ABM), System Dynamics (SD), Discrete Event Simulation (DES), Bayesian Networks (BN)
Optimization (O)	Linear, Nonlinear, Multi-objectives, Mixed Integer, and Dynamic Programming
Spatial modeling (SM)	GIS
Multicriteria decision analysis (MCDA)	TOPSIS, AHP, Fuzzy TOPSIS, Fuzzy AHP
Graph theory (GT)	Centrality measures, Dijkstra
Text mining	
Others	Visualization, Petri net, Cognitive maps
- Data sources: We identified three main data sources: primary, secondary, and hypothetical; see Table 2.

**Table 2:** Data sources definitions.

Data source	Definition
Primary	Data were derived by the researchers from interviews, surveys, observations, designing, and building databases with third parties.
Secondary	Data come from previous research, social media and newspapers, publicly available reports, publicly available data such as census data, and data owned by the local authority of a specific community (not publicly available).
Hypothetical	Simulated or generated data.
Both	Means both secondary and primary data.
- Disaster phase: The publications are classified based on the four phases of the disaster life cycle: mitigation, preparedness, response, and recovery [28].
- Hazard types: Cover different types, such as earthquakes, floods, tsunamis, landslides, hurricanes, extreme weather, and multiple hazards.

**Fig. 3:**
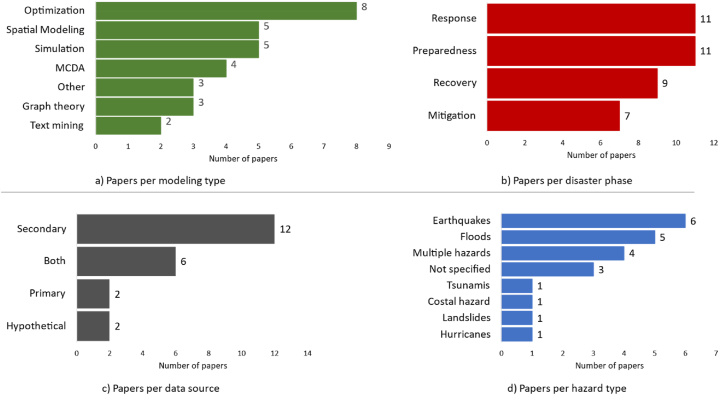
Distribution of papers across a) modeling type, b) disaster phase, c) data source, d) hazard type. Note that for sub-figures a and d, the sum of the papers in the graph is more than 23, as some papers cover different modeling types or phases respectively.

Table 3 describes each of the identified papers applying the previously mentioned criteria. Fig. 3 shows the number of papers covering each of the characteristics (Fig. 3-a covers the modeling techniques, Fig. 3-b covers the disaster phase, Fig. 3-c covers the data sources, and Fig. 3-d covers the hazard type). Almost 35 % of the papers combine different modeling methodologies. Some of them combine two different techniques, simulation and optimization, for example in Ref. [29], and others utilize two techniques from the same category (MCDA), fuzzy AHP with fuzzy TOPSIS [30]. Optimization techniques rank first in the modeling techniques used (Fig. 3-a). 75 % of the papers that use optimization techniques cover the recovery phase of the disaster life cycle. 80 % of the publications using Spatial Modeling cover the preparedness phase (See Table 4 for the distribution of modeling types across disaster life cycle phases). Furthermore, many of the identified papers present a general model applicable to any type of disaster; however, the choice of case studies guides the application to a specific disaster type. Some models cover multiple disaster phases. Depending on the time scale of the decision, the models could be assigned to different phases. For example, if the decision support system helps in the restoration of power services this will be assigned to the response phase, but if the restoration is related to housing, this will be assigned to the recovery phase. Furthermore, most of the research is conducted using data from a developed country, specifically the United States. In the next subsection, we are going to summarize the papers categorizing them by modeling type.

**Table 3:** Characteristics of the selected decision support tools.

#	Hazard type	Disaster phase	Modeling type	Data source(s)	Reference	Place (Spatial level)	Purpose
1	Multiple	M, R	S, GT	Secondary	[31]	USA (City)	Manage community-level infrastructure by considering different mitigation and response measures.
2	Floods	All	GT, Others	Primary	[32]	UK (Town)	Identify and prioritize the flood resilience intervention actions and strategies.
3	Multiple	M, RC	O, S	Both	[33]	Kenya (City)	Assist long-term planning process by selecting resilience-related projects considering their synergies.
4	Costal	P, M	MCDA	Both	[34]	Canada (Small Island)	Assess how coastal adaptation decisions impact community resilience.
5	Earthquakes	RC	O	Both	[35]	USA (County)	Identify recovery actions after an earthquake.
6	Floods	R, RC	S	Secondary	[36]	Philippines (City)	Testing various resilience policies on the household and local government levels.
7	Earthquakes	RC	O	Secondary	[37]	USA (County)	Test the impact of humanitarian and operational considerations on optimal infrastructure restoration decisions while considering the effect of pre-disaster decisions (ex. resource allocation).
8	Not specified	P	SM	Primary	[38]	USA (County)	Build a shared knowledge repository and resource allocation model for preparedness activities.
9	Earthquakes	RC	O	Secondary	[39]	USA (County)	Find optimal restoration decisions of interdependent networks which are controlled by decentralized agents.
10	Floods	P	MCDA	Both	[40]	UK & Germany (Catchment area)	Rank flood risk management alternatives against specific objectives.
11	Floods	P	SM	Secondary	[16]	France (City)	Build a spatial decision support system guided by resilience assessment measures.
12	Multiple	R	O, SM, MCDA	Secondary	[41]	Haiti (Country)	Find optimal shelter locations following a risk minimization and service availability maximization approach.
13	Earthquakes	RC	O	Secondary	[42]	USA (City)	Allocate resources to building retrofits after disasters.
14	Floods	P, R	SM, Others	Secondary	[43]	Canada (City)	Test the impact of different flood adaptation strategies on resilience.
15	Not specified	All	Text mining	Secondary	[44]	Portugal (--)	Build a chatbot for real-time information about disasters.
16	Landslides	M, P, R	SM, Others	Secondary	[45]	Italy (Municipality)	Create a spatial data infrastructure to guide the decision-makers through the procedures related to emergency management.
17	Multiple	P	Text mining	Secondary	[46]	USA (community)	Link community needs to cover climate risks with suitable federal plans for climate change adaptation
18	Not specified	All	O	Hypothetical	[47]	–	Minimize the cost of infrastructure interventions, considering different types of interdependencies among the infrastructures.
19	Earthquakes	R	GT*	Secondary	[48]	Papua New Guinea (Country)	Develop a near real-time information system for delivering humanitarian aid in response to a disaster.
20	Hurricanes	R	S, O	Hypothetical	[29]	USA (County)	Determine the optimal repair schedule of the affected civil infrastructure considering their interdependencies with social infrastructures.
21	Tsunamis	R	S	Both	[49]	Chile (Municipality)	Facilitate the evacuation of individuals after a tsunami.
22	Earthquakes	P	MCDA*	Both	[30]	Nepal (District)	Build a group decision support framework for selecting shelter locations.

Disaster phases: Mitigation (M), Preparedness (P), Response (R), Recovery (RC). Modeling type: Simulation (S), Optimization (O), Spatial Modeling (SM), Multi-Criteria Decision Analysis (MCDA), Graph Theory (GT).

**Table 4:** Percentage of papers applying a specific modeling type VS disaster phase.

Modeling type	Mitigation	Preparedness	Response	Recovery
Optimization	25 %	13 %	38 %	**75 %**
Spatial Modeling	20 %	**80 %**	**60 %**	0 %
Simulation	40 %	0 %	**80 %**	40 %
MCDA	25 %	**75 %**	25 %	0 %
Text Mining	50 %	**100 %**	50 %	50 %
Graph Theory	**67 %**	33 %	**100 %**	33 %

### 2.3 Results based on modeling technique

In this section, we present the results of our analysis, grouping the various studies according to their modeling techniques. We'll cover six modeling techniques outlined in Table 1.

1) Optimization: focuses on finding the best solution to a problem by considering different factors and constraints, often using mathematical algorithms to identify the most efficient or effective option [50].
2) Spatial Modeling: Utilizes geographic information systems (GIS) and other tools, to capture spatial relationships, analyze, predict, and visualize how geographic factors influence processes or decisions [51].
3) MCDA: helps decision-makers weigh different factors, evaluate alternatives, and identify the most suitable option based on their priorities and objectives when faced with multiple, sometimes conflicting, criteria [52].
4) Simulation: creates a digital model to mimic real-world systems or processes, allowing for experimentation and analysis in a controlled setting [53].
5) Graph Theory: studies relationships between objects by representing them as nodes and edges in a graph [54].
6) Text Mining: extracts knowledge and insights from large amounts of textual data using computational methods.

Additionally, there is a subsection covering the studies that used multiple modeling techniques.

#### 2.3.1 Optimization

The papers covered in this section utilize optimization and mathematical modeling techniques to build decision support systems for resilience [35]. The first study in this category [35] proposes a mathematical model for the recovery phase. The paper follows a sequential discrete optimization approach to identify the near-optimal actions such as assigning resources to repair work after an earthquake to recover an electrical power network. The proposed method considers all interdependencies and cascading effects among different components within the recovery process rather than just the network level. Some of the data in the model are derived from other publications and expert opinions.

Another paper that considers the interdependencies between different components is [37]. However, the authors in this paper focus on the relationships between the physical and socioeconomic systems. Recovery and restoration decisions are not only guided by operational costs, but also by interests of the different community actors, existing policies, and pre-disaster decisions. This paper suggests a model for optimizing the recovery of critical infrastructure. The model takes into account all the factors mentioned earlier and incorporates them into both the objective functions and the constraints of the model. The formulation is presented as an interdependent network design problem (INDP) with a weighted multi-objective. The main goal of the proposed framework is not the resulting optimal values *per se*, but providing a platform for policymakers to analyze and assess the impacts of the different operational and socioeconomic configurations on resilience, allowing them to make informed decisions and propose new standards based on solid objective analysis. The model was applied to data from a specific network operator in Shelby County, Tennessee, USA [39]. build upon the core model proposed in Ref. [37]. But instead of a single agent responsible for the whole network, they consider multiple agents in the infrastructure network making decisions to minimize the costs in their respective network layer. They assume a lag in communication between the different agents, and each agent needs to predict the decisions of the others and make “a judgment call” to act based on these predictions. The model was applied to the same data as in Ref. [37].

The authors in Ref. [42] built a multi-objective optimization model that can also be used in the recovery phase of a disaster. The model focuses on the building retrofit decisions that happen after a disaster, more specifically how to allocate the available limited resources. The model has two conflicting objective functions: (1) minimizing the direct economic loss, and (2) minimizing the total population dislocation. The model was solved using the ϵ method and was applied to the Centerville virtual community. In the last paper in this subsection, a multi-stakeholder optimization strategy for managing infrastructure interventions is proposed [47]. The mathematical model is formulated as a mixed integer nonlinear optimization problem, minimizing the cost of intervention. The model considers the interdependencies between infrastructures, whether physical, functional, or geographical. The authors also considered the idea of grouping the maintenance work, and how some maintenance work cannot be moved in time because it depends on other aspects. The model was tested on hypothetical/simulated data and solved using an integer genetic algorithm. The model's application in a specific disaster phase is determined by the type of intervention required, such as elimination, maintenance, upgrading, or renovation.

From this discussion, optimization techniques could be highly effective in building resilience decision support, as they offer a range of models that could capture multiple aspects of the problem at hand such as the interdependencies between different components and community preferences.

#### 2.3.2 Spatial modeling

The publications discussed in this subsection use the Geographical Information System (GIS) as the main building block of their decision support frameworks. [38], proposes a prototype decision support system that serves as a shared knowledge repository and resource allocation model; the framework is supposed to be used in the preparedness phase. They worked with the Allegheny County Emergency Services (Pennsylvania) and other local emergency organizations to build an accurate geodatabase of all emergency response facilities in the county. Additionally, they developed two types of models: (1) defining service areas based on estimated travel times, and (2) selecting the optimal location of service units to maximize the demand coverage in disaster locations. The service area module aids in the analysis of the current resource allocation situation. The decision about where to effectively place/locate new resources or relocate existing ones is handled by the optimal location selection module. The prototype was evaluated positively by emergency practitioners through a focus group.

Another geographical decision support system was proposed in Ref. [16]. The article aimed at operationalizing the concept of resilience by combining geographical modeling with resilience indicators. The framework follows two main building blocks: first, defining and measuring resilience through a set of different indicators, and second, using a GIS software system to create a spatial decision support system that reflects the resilience values across different geographical locations and time frames. The model was applied in Avignon, France, using publicly available data sources such as INSEE and SIRENE. Moreover, the model was developed in collaboration with the local stakeholders of the Avignon area to ensure the usability of the tool.

Spatial modeling enables the matching of resilience indicators with their corresponding geographical locations. This provides a detailed picture of the resilience status in a specific location. Furthermore, the visual nature of spatial modeling makes it easier to understand when compared to only numerical or textual data. Furthermore, because of its reliance on GIS, spatial modeling captures how the physical world works, making the model more reliable.

#### 2.3.3 Multicriteria decision analysis

The papers covered in this subsection use multicriteria decision analysis techniques to support resilience decision-making. The first one [34] presents a framework for assessing coastal adaptation decisions (protect, accommodate, retreat, and do nothing) in a Canadian coastal community. The Analytical Hierarchy Process (AHP) was used to rank the various adaptation decisions based on four main pillars: environmental, cultural, economic, and social. The framework also considered historical storm impact data associated with the four identified pillars. Different community stakeholder groups were involved in the decision-making process, reflecting their preferences using the scores of the AHP. The authors emphasized the AHP's suitability for these types of multicriteria group decision-making models because it can handle both qualitative and quantitative data and is simple to use. Furthermore, the researchers point out the issue of data availability, stating that because there is no formal community reporting mechanism for storm impacts, they had to rely on newspapers and publicly available data that present aggregated values. Another publication that highlights the importance of participatory modeling and takes into account the different perspectives of community stakeholders is [40], but in this case, they use Technique for Order Preference by Similarity to Ideal Solution (TOPSIS). The paper uses TOPSIS to rank the different flood risk management alternatives against five objectives in two catchment areas in Germany and the UK. The framework uses publicly available data to determine, for example, the values of the magnitude of flooding and expert opinions to reflect the feasibility of the implementation of one alternative.

The last paper in this category [30] combines both TOPSIS and AHP techniques in a group decision support framework for the problem of emergency shelter allocation. The authors first identify the criteria for selecting a shelter location through a literature review. A focus group with emergency experts then cleans up the list by eliminating or adding new criteria. After that, they use fuzzy AHP – based on experts’ input – to prioritize these criteria. Next, the authors introduce the candidate sites of the shelters and apply fuzzy TOPSIS to select the best shelter location among the different alternatives. The use of fuzzy logic here is due to the difficulty and uncertainty that always accompany the evaluation of qualitative factors, as well as the fact that disaster decisions are typically made with limited information.

We can conclude from the three studies that both TOPSIS and AHP are appropriate for group decision-making and capturing the qualitative aspects of the problem being examined. Furthermore, TOPSIS may be preferable to AHP when there are many alternatives to choose from because AHP's pairwise comparison among many alternatives is a complex process [30].

#### 2.3.4 Simulation

The two studies in this section use different simulation techniques to support resilience decision-making. The first paper [49] uses Agent Based Modelling (ABM) to study the micro behavior of the entities of the system using a bottom-up approach, while the second one [36] uses system dynamics, highlighting the macro behavior of the system in a more top-down approach. In Ref. [49], the authors developed an ABM to assess the suggested topological modifications of the street network in Talcahuano, Chile, to facilitate evacuation after a tsunami; they used evacuation time as an indicator to compare the different settings. The modeling approach proves to be useful in testing different urban design configurations. The authors used different data sources to build the model: interviews with emergency stakeholders, an on-site survey to investigate the current urban conditions, and census data for demographics.

The second study [36] aimed to provide a decision support tool for testing various community resilience policies. Resilience is measured as the level of household assets and local government assets; any disruption could cause a loss of these assets, hence a loss of system performance. The data sources for this study come from a variety of sources, including government reports and other research papers. The paper also suggests involving stakeholders in the model-building process and identifying key variables that reflect what is important to them; in this way, the model-building process could also serve as a stakeholder engagement approach.

Utilizing simulation techniques enables in-depth analysis of system behavior and investigation of the underlying structures of interactions. They also provide a useful platform for testing the impact of various policies and decisions in advance.

#### 2.3.5 Graph theory

In [48] the authors leverage graph theory techniques to develop a near real-time information system for delivering humanitarian aid when a disaster happens. The information system captures the status of the roads and entry points in a specific area after a disaster. They first use OpenStreet Maps to obtain the map information and then use other information sources to reflect the overall status of the crisis. This information covers the status of the roads and infrastructure, travel times, and logistic operations^1^; humanitarian data covered in ReliefWeb reports^2^; and other demographics and disaster-related information from the Humanitarian Data Exchange Portal (HDX)^3^. Then, they apply centrality measures and the Dijkstra algorithm, and produce visualizations for the decision-makers to help determine how the aid could be distributed. Centrality measures are part of network analysis techniques, and they assess how "important" a specific location in an area is based on its connections to other locations and roads [54]. While the Dijkstra algorithm is used to find the shortest path between two locations in an area (road network) [55]; the users of the model could run the algorithm based on either the travel time, or the length of the road between the two locations, or on both. The model was applied using data from an earthquake that hit Papua New Guinea in 2018. Two response professionals reviewed the model, emphasizing its usefulness in terms of saving time and quickly establishing an assessment of the situation.

#### 2.3.6 Text mining

Here, we have a different type of modeling; the two papers presented in this section build upon Artificial Intelligence technologies, namely text mining techniques. The first one [44] proposes a chatbot (DisBot) that can be used by both emergency responders and citizens. The chatbot builds upon Twitter data to obtain real-time information about disasters and then shares this information, together with recommendations and official procedures, with the end user upon request. The chatbot was evaluated by civil protection specialists, who highlighted the importance of the chatbot but were concerned about its adoption; one of the suggestions to face this challenge was to embed the chatbot into the existing municipality mobile application. The second paper in this category [46] presented an automated decision-support tool (ASAP) to link community needs to cover climate risks with suitable federal plans for climate change adaptation. The authors used vector space modeling to identify semantic similarities between documents (community needs and federal plans).

Text mining techniques save decision-makers and emergency responders time and effort. They enable the identification of disaster information through social media listening and the rapid identification of suitable plans.

#### 2.3.7 Combined modeling

In this subsection, we discuss research that combines different modeling techniques to build decision support systems. The first paper [32] examines flood resilience by combining fuzzy cognitive mapping with centrality measures to identify and prioritize flood resilience intervention actions and strategies. The flood resilience interventions are based on the flood resilience measurement framework (FRMC (https://floodresilience.net/frmc/)), which covers all the phases of the disaster life cycle. Following a participatory modeling approach (through focus groups and interviews) the study collected data on the importance of the different flood interventions according to each of the stakeholders and the impact of these interventions on each other. These data were used to build a fuzzy cognitive map, capturing the causal relationships between the different types of interventions. Centrality measures were then applied to prioritize the interventions based on their impact. The study was conducted with stakeholders in Lowestoft, UK. The methodology introduced in this paper considers the mental models of the various stakeholders in the community and builds upon their knowledge to support context-specific decision-making. Moreover, centrality measures provide a suitable tool to capture and rank the interdependence between the different interventions.

Another study that utilizes graph theory techniques as part of its combined modeling is [31]. However, in this study, the main focus is simulation techniques, while graph theory techniques present a small component in the model. The study proposed a decision support system for community-level infrastructure management. It considers the effect of various mitigation and response measures (e.g., providing different sources of water supply or other shelter locations) on infrastructures such as buildings, power, and water systems. The proposed system allows the stakeholders to choose different types of infrastructures, hazards, mitigation and response measures, and resource availability levels. Monte Carlo simulation (MCS) is used to generate uncertainties in the damages, repair time, and financial losses. Breadth-first search is used as part of the simulation to determine the connectivity of the different infrastructures to propagate the damages and impacts across the connected infrastructure. Breadth-first Search is a graph traversal algorithm employed to systematically explore all nodes in a graph [54]. MCS results are used to feed a Bayesian network which calculates the system resilience. Resilience is defined as the joint probability of achieving rapidity and robustness performance goals, where rapidity is defined as “the restoration time” and robustness is defined as “acceptable decrease in the immediate post-event performance of infrastructure systems” The framework was applied in Seaside, Oregon, USA, using publicly available data.

Another set of publications utilizes spatial modeling combined with other modeling techniques. For example, in Ref. [43], the authors developed a web-based tool called ResilSIM, which supports decision-making by measuring the urban system's resilience to floods. The tool uses physical and socioeconomic flood resilience metrics to measure the system's resilience. These metrics are associated with geographical locations using spatial modeling. Flood inundation maps are produced using hydraulic modeling. Using these maps and publicly available data, the baseline resilience metrics are calculated considering the different locations. Users (decision-makers) can then test the impact of different adaptive capacities by changing the model configuration through a friendly user interface; these capacities can be reactive (implemented during the floods) such as the evacuation of people, or proactive (implemented before the floods) such as maintaining the drainage infrastructure. Based on the new configurations, the system calculates the resilience measures again, and the user can compare the performance of the system before and after applying the adaptation actions. The tool works in near real-time and was evaluated using data from London, Ontario, Canada.

The authors in Ref. [45] propose a Spatial Data Infrastructure (SDI) for emergency management in Italy. The SDI consists of multiple parts: a heterogeneous data repository; a GIS system for geospatial visualization; a Volunteered Geographic Information (VGI) for collecting data about the situation at hand from volunteers and generating reports based on this data; an engine to cross-validate the VGI with the organizational data available; a data querying engine; a decision support system that includes Petri Nets to guide the emergency responders step by step through the response process following the already existing protocols; and finally, a communication service using emails, Skype and SMS. The main contribution of the proposed system is the integration of all these different functionalities. It also combines organizational data (e.g., maps, resources, personnel) and protocols and nonorganizational data (e.g., research and data provided by volunteers) in one place, which makes the system useful for multiple users such as academics and emergency experts. Moreover, the system could be used for training purposes, as there is a log functionality that records all the actions and the person who implemented them; hence, it can be used to identify what went wrong, how the operator acted, and the lessons learned. The SDI was implemented and tested with a hypothetical metrological emergency with the Regional Office of Civil Protection in Northern Italy. Despite the advantages of the system, according to the Civil Protection Department, it still lacks the functionality to handle concurrent risks.

The researchers in Ref. [41] use spatial multi-criteria decision analysis techniques to facilitate shelter planning decisions against multiple hazards. The proposed framework incorporates two phases, the first to determine the suitable shelter sites and the second to select the most suitable sites. To identify the potential shelter sites, the framework considers two objectives: minimizing risk and maximizing service availability. AHP was used to determine the weights of each of the objective functions and the weighted linear combination feature in ESRI ArcGIS software was used to integrate the objectives and the different attributes associated with them. In the second phase, the location-allocation optimization module in ESRI ArcGIS software was utilized to find the optimal shelter location considering the population density in the different locations. The framework was validated in the Haiti case using publicly available data. Although one of the main purposes of this study is to build upon the currently available data to enhance resilience everywhere, the authors highlight that there is still a problem with data availability in low-income societies.

Another publication that utilizes optimization techniques but, in this case, combined with simulation modeling is [29]. The paper proposes the Civil Restoration with Interdependent Social Infrastructure Systems (CRISIS) decision support system. CRISIS considers the dependencies between civil infrastructure (e.g., water, power, and transportation networks) and social infrastructure (e.g., police, hospitals, and commercial services). The system seeks to find the optimal schedule for the restoration and repair of damaged civil infrastructures considering stakeholders’ priorities for the disrupted social infrastructures. By optimizing the repair of the civil systems, the model maximizes the functionality of social systems. The model output is an optimal schedule highlighting which components should be fixed, by whom, and when. The model includes two parts: the first uses simulation modeling (Monte Carlo Simulation, and the simulation component in HAZUS-MH software) to simulate the disruption of the infrastructure systems; the second uses an optimization model to generate the optimal schedule. The model was applied using hypothetical data for a virtual community in the USA so as not to expose sensitive information about the vulnerabilities in the real infrastructures.

The second study that applies both optimization and simulation is [33]. The authors proposed a mathematical model to support decisions on project implementation to improve the mitigation and long-term recovery phases of resilience while considering the synergies between the various projects (ex. build sea walls, develop early warning systems). The model is built as mini-max multi-objective linear programming. Community preferences were incorporated into the model as part of the constraints and objective functions. In addition to community preference data, the authors used expert opinions to feed a simulation model to estimate the severity and impacts of the investigated hazards. Additionally, they utilized publicly available data such as estimated causalities of hazards that come from Kenyan governmental reports to define the model parameters.

## 3 Discussion

In this section, we delve into the key findings that emerged from the literature review. This section will analyze and discuss the current challenges identified within the existing research. Additionally, it will propose practical guidance for selecting an appropriate modeling technique tailored to specific community resilience contexts.

### 3.1 Data

So far, this paper has emphasized that creating a resilience model is a complex and data-intensive process that involves multiple aspects and utilizes both quantitative and qualitative data. However, the quantity and type of data depend on the modeling technique employed. For instance, spatial modeling requires large datasets that include not only resilience-related information but also geographical information, such as longitudes and latitudes, road networks, and building locations. Additionally, spatial modeling incorporates maps of seismic hazards and tornado paths, providing a precise representation of real-world hazards. Graph theory techniques also use spatial data to determine shortest paths and maximum flows, among other things.

Furthermore, graph theory models utilize dependency data that could be derived from previous events or provided by experts. Bayesian models also utilize data that describe the dependency between different events or risks. Additionally, Bayesian models need probabilistic data to work. Monte Carlo simulation also depends on probability distributions. Text mining models, on the other hand, are built upon huge datasets, but unlike the structured data used in the previously mentioned models, here the data is unstructured.

Optimization models are usually less data-intensive than the other modeling techniques, and they allow for including stakeholders’ opinions in the form of model parameters or coefficients. MCDA models are more dependent on expert opinions than other types of modeling, such as graph theory, because the main idea is to rank alternatives or criteria that directly reflect stakeholder preferences.

### 3.2 Modeling technique

Beyond the usage of the data, optimization models offer a comprehensive decision support system that considers multiple aspects of a problem and stakeholders' perspectives while providing an optimal/exact solution. They effectively capture the synergies between the various actions that constitute a decision. Spatial modeling, on the other hand, allows for the integration of multiple functionalities, including geolocation data, visualization capabilities, graph theory algorithms, and optimization models.

Simulation modeling, particularly agent-based modeling (ABM), provides a better understanding of complex resilient systems and predicts their future state based on the initial state captured by certain parameters. Monte Carlo simulation is suitable for simulating disaster damage, while Bayesian models are useful for modeling probabilistic events and cascading impacts of emergencies from one part of the system to another. Network analysis techniques, specifically centrality measures, are appropriate for capturing the deterministic interdependencies between different parts of the system. Additionally, centrality measures are suitable for ranking highly interconnected resilience-related factors.

MCDA methods are effective in capturing qualitative aspects of a problem and facilitating group decision-making, while properly configured text mining techniques minimize the need for decision-maker intervention. However, retraining and data updating are still necessary. For instance, a disaster chatbot operates automatically, whereas an optimization model requires manual activation each time a decision must be made.

### 3.3 Relationship between modeling technique and disaster phase

Analyzing the information included in Table 4, we can conclude that some modeling techniques are used in association with specific disaster phases more than others. For example, 75 % of the papers that applied MCDA techniques used them in the preparedness phase. This is due to the nature of the MCDA techniques; they are used for group decision-making which requires a long time to collect the information and perform the analysis. These techniques are not designed for a prompt response and are therefore used for preparedness where there is time to compare alternative plans and policies.

Spatial modeling is also used in the preparedness phase (80 % of the papers) and in the response phase (60 %). The integration capabilities mentioned previously, indicate that spatial decision support systems are one of the most suitable platforms for resilience. In the response phase, as decision-makers can see the situation at hand by visualizing the physical world, they can use spatial modeling to distribute resources, identify blocked roads, find alternatives, and run shortest-path algorithms to reallocate the resources or distribute humanitarian aid. While in the preparedness phase, spatial DSSs enable the users to make more proactive or strategic decisions. For example, the system visualizes the maps and demand distributions, allowing the users to run optimization models for location-allocation problems such as finding the optimal location for shelters.

Moreover, simulation models are used to address the response phase by imitating the behavior of the system and individuals during a disaster. Simulation models are employed before the event, but they are usually built to address what should happen during the response phase.

Graph theory techniques, particularly tree algorithms, and shortest path algorithms, are also used in the response phase. They are appropriate in this phase because all connected infrastructures or roads form a network in which the disaster's cascading effects should be analyzed (using tree algorithms) and the shortest distance to distribute resources and aid should be calculated.

Lastly, optimization techniques are utilized in the recovery phase, since they are mostly used for retrofitting decisions. These types of decisions are subject to resource availability making optimization models the most suited for these decisions.

### 3.4 Stakeholders’ engagement in DSS building

Our review identified three primary approaches to stakeholder engagement in the development of the various DSSs. The first approach utilizes stakeholders as data sources. This can involve soliciting data and opinions directly from experts or community members [32,34,39]. Alternatively, community opinions and information shared on platforms such as Twitter (currently named X) can also serve as a source of data for building chatbots [44].

The second approach involves stakeholder participation in problem identification, model building, and system development. Studies like [40] cover how stakeholders participated in a series of workshops to cover the flood situation in their area, starting from problem identification and extending to ranking potential solutions. Additionally, stakeholders can be included in defining system functionalities and building the system itself. For example, in Ref. [16], the authors directly involved local utility managers and county officials in the creation and refinement of the proposed model. This approach ensures the tool's relevance and practicality for stakeholders, reflecting real-world applicability.

The third approach focuses on incorporating stakeholder preferences into the DSS model itself. This can be achieved by reflecting these preferences as parameters for the mathematical models used within the DSS. Examples include [33], where community preferences were integrated into the model as constraints and objective functions, and [37], where preferences from different actors were incorporated as model constraints to reflect the potential impact of regulatory measures on recovery processes and associated costs.

### 3.5 Challenges

The development of DSS for enhancing and supporting community resilience is hindered by several challenges. One of the main challenges is the complexity and variability of the data and information needed for DSS to make accurate predictions and recommendations. This data often comes from multiple sources with varying levels of quality and accuracy, making it difficult to integrate and use for decision-making purposes. The publicly available data that come from secondary sources such as census reports are not available in all countries at the same level or do not include the same attributes. In less developed countries, it is harder to access this type of information, making it difficult to apply the same models across different locations [41]. Moreover, sometimes we need to use proxy variables to represent “soft” variables, for example using the amount of extra food people have at their houses to reflect their preparedness level.

Collecting data from experts or community stakeholders is also a challenging task, as it requires finding the right representative individuals, securing their agreement to participate in the study, and conducting the data collection.

Another data-related issue is data privacy. Exposing sensitive information about a specific community could pose a risk to the security and well-being of its residents. This information can include details about critical infrastructure, emergency response plans, and sensitive locations, all of which can be used by malicious actors to cause harm [22]. To avoid these problems, it is better to anonymize the data or use hypothetical data (such as [29]) to ensure that sensitive information remains protected.

Integrating the developed DSS into emergency organizations and their daily operations is another challenge [37]. Simply involving some emergency personnel in the development of the DSS does not guarantee its acceptance nor its use in the routine procedures of emergency organizations. This integration depends not only on the proposed DSS effectiveness but also on budget availability and emergency organizations' willingness to adopt new systems.

Furthermore, the development of DSS for resilience often requires interdisciplinary collaboration and a deep understanding of both technical and societal issues, making it difficult to find individuals with the necessary expertise and skills [42,56].

Building a DSS to handle real-time changes and unexpected events can be challenging due to the dynamic, unpredicted, and complex nature of resilience-related situations. This requires a high degree of flexibility and agility in the design of the DSS so that it can adjust to unexpected changes in the environment rapidly.

### 3.6 Guidelines for selecting a modeling technique

Based on the analysis of the papers described in section 2.3, we are proposing some guidelines designed to help in the selection of an appropriate DSS for community resilience based on a specific context. These guidelines aim to provide researchers and decision-makers with clear and concise recommendations for building a DSS. The guidelines consider three aspects of the problem context: the purpose of the DSS, the extent of the data intensity, and the promptness of the decision.

The purpose of the DSS means the goal or the objective of the DSS. We identified three main purposes that are common among the different publications: resource allocation, prioritization, and policy testing. Resource allocation is related to the assignment and distribution of different resources (financial, human, machinery) among various tasks related to community resilience. Prioritization is related to ranking different alternatives, whether strategies, policies, locations for shelters, and so on. Finally, policy testing is related to verifying a plan or an action and assessing its functionality and effectiveness.

The data intensity of a modeling technique refers to the amount of data required for the technique to generate accurate and reliable results. Some modeling techniques, such as text mining algorithms, require large amounts of data to train the model and produce accurate predictions. These techniques are high in data intensity. Other techniques, such as optimization models, may require less data and be considered low in data intensity. The availability, cost, and effort in collecting and processing data will impact the feasibility and effectiveness of the modeling technique. Therefore, the data intensity is an important consideration when selecting a modeling technique for a particular application.

Finally, the promptness of the decision is the extent to which the decision is made quickly. High prompt decisions need little time to be made. For example, once a text mining model has been trained, it is capable of responding to requests in a matter of seconds. However, a simulation model will take some time to run, produce accurate results, and then analyze the results.

Table 5 shows the distribution of the modeling techniques among the three mentioned criteria, accompanied by a brief description of the modeling techniques and their strengths and limitations. Note that the modeling techniques could be used for purposes other than those highlighted in the graph, but these assignments are based on many of the reviewed studies.

**Table 5:** Application of modeling techniques in community resilience.

Modeling Technique	Brief Description	Data Intensity	Promptness	Purpose	Strengths	Weaknesses
Text Mining	Analyzes large volumes of text data (e.g., social media, reports) to extract insights and patterns.	High	High	Resource allocation - Prioritization (indirectly)	Can reveal hidden patterns and sentiment in large datasets.	Require pre-processing and may not be generalizable to other contexts.
Spatial Modeling	Creates geographic visualizations to analyze spatial relationships and patterns.	Moderate to High	High	Resource allocation	Provides a clear visual representation of risks and resource allocation.	Data quality and availability can be a challenge and require specialized software.
Graph Theory	Models systems as interconnected nodes and edges to analyze relationships and network dynamics.	Moderate to High	High	Prioritization	Effective for analyzing network connectivity and dependencies.	May not capture all system complexities and may be difficult to interpret for non-experts.
Simulation	Creates a virtual representation of a system to model its behavior under different scenarios.	Moderate	Low	Policy analysis	Allows for experimentation in a safe environment, and can incorporate various factors and complexities.	Time-consuming to develop and validate.
Optimization	Uses mathematical algorithms to find the best solution based on defined criteria.	Moderate	High	Resource allocation	Provides efficient solutions for specific problems.	May not account for all real-world complexities, limited to problems with well-defined objectives.
MCDA	Supports decision-making with multiple conflicting objectives by structuring preferences and evaluating alternatives.	Low to Moderate	Low	Prioritization	Provides a systematic approach to decision-making (and group decision-making).	Time-consuming, and requires careful definition of objectives and weights.

## 4 Conclusions

DSS enables the operationalization and enhancement of resilience. This article examines the role of DSS in enhancing community resilience by focusing on its various applications. Through a comprehensive literature review, we analyzed the utilization of DSS in different phases of the disaster management cycle and identified the main modeling techniques utilized. Our findings indicate that the focus of DSS applications is primarily on disaster response and preparedness, rather than recovery and mitigation. Optimization models were the most commonly used techniques to develop a DSS. As for the use of data, a majority of the studies reviewed were based on secondary data sources. Moreover, some of the publications mentioned consider stakeholder opinions in their models, whether through participatory modeling [32,40] or as parameters for mathematical models [33], however, none of these studies focus on modeling stakeholders’ interactions in more detail.

Despite the positive impact that DSSs have on community resilience and disaster management, some limitations remain. These include issues around data availability and privacy which need to be addressed. Additionally, the unexpected nature of disasters and the need for real-time actions pose challenges to the development and effective use of DSS in a resilient context. Furthermore, the application and the integration of DSS in day-to-day disaster management decisions need to be analyzed.

A potential area for future work could be exploring how DSS could be utilized for the recovery and mitigation phases of disaster management. Additionally, exploring the incorporation of real-time data sources and IoT technologies could improve DSS responsiveness and effectiveness in dynamic disaster contexts, addressing one of the identified challenges of handling real-time changes. Moreover, future research could delve into the concept of building DSS that prioritizes community engagement. This could involve exploring methods to facilitate stronger interactions and collaboration among various community stakeholders, ultimately fostering a more resilient and prepared community.

This study provides a comprehensive summary of the state of the art of DSS applications to community resilience and a guideline for choosing the most appropriate modeling technique for a given context. The goal of the study is to help decision-makers and researchers build effective decision-support systems and frameworks that enhance community resilience while taking into consideration the current challenges.

## Funding

This research was supported by the European project ENGAGE funded from the 10.13039/501100007601European Union’s Horizon 2020 research and innovation program under grant agreement no. 882850

## Data availability

No data were used for the research described in the article.

## CRediT authorship contribution statement

**Sahar Elkady:** Writing – review & editing, Writing – original draft, Investigation, Formal analysis, Conceptualization. **Josune Hernantes:** Writing – review & editing, Supervision, Conceptualization. **Leire Labaka:** Writing – review & editing, Project administration, Funding acquisition, Conceptualization.

## Declaration of Generative AI and AI-assisted technologies in the writing process

During the preparation of this work the author(s) used ChatGPT and Google Bard in order to paraphrase some of the text to improve its readability. After using this tool/service, the author(s) reviewed and edited the content as needed and take(s) full responsibility for the content of the publication.

## Declaration of competing interest

The authors declare that they have no known competing financial interests or personal relationships that could have appeared to influence the work reported in this paper.

## Footnotes

1 Logistics Cluster serves as a central hub for critical information in times of crises. This includes up-to-date logistics updates, details on road conditions and access points, and essential documents like meeting minutes and procedures for various countries and disaster types (https://logcluster.org/).

2 ReliefWeb is a humanitarian information service provided by the United Nations Office for the Coordination of Humanitarian Affairs (OCHA) (https://reliefweb.int/).

3 HDX is the data-portal of OCHA, providing a rich set of geographical, demographic and disaster-related information (https://data.humdata.org/)
